# Persistently increased frequency of diabetic ketoacidosis in new-onset type 1 diabetes in Polish children: nationwide analysis 2019–2022

**DOI:** 10.3389/fendo.2026.1861694

**Published:** 2026-06-29

**Authors:** Arkadiusz Michalak, Barbara Pernak, Jędrzej Chrzanowski, Iwona Pietrzak, Iwona Beń-Skowronek, Artur Bossowski, Agata Chobot, Maria Bednarska, Katarzyna Dżygało, Wojciech Fendler, Barbara Głowińska-Olszewska, Martyna Górnicka de Almeida, Anita Horodnicka-Józwa, Katarzyna Jakubek-Kipa, Przemysława Jarosz-Chobot, Anna Kącka, Katarzyna Marcinkiewicz, Artur Mazur, Małgorzata Myśliwiec, Joanna Nazim, Barbara Wasyl-Nawrot, Elżbieta Niechciał, Anna Noczyńska, Ewa Rusak, Sebastian Seget, Monika Seifert, Elżbieta Skotarczyk-Kowalska, Anna Skowronek, Lidia Groele, Paulina Wais, Mieczysław Walczak, Anna Wołoszyn-Durkiewicz, Marta Wysocka-Mincewicz, Agnieszka Zubkiewicz-Kucharska, Agnieszka Szadkowska

**Affiliations:** 1 Department of Pediatrics, Diabetology, Endocrinology and Nephrology, Medical University of Łódź, Łódź, Poland; 2 Department of Biostatistics and Translational Medicine, Medical University of Łódź, Łódź, Poland; 3 Department of Pediatric Endocrinology and Diabetology, Medical University in Lublin, Lublin, Poland; 4 Department of Pediatrics, Endocrinology and Diabetology with Cardiology Unit, Medical University of Białystok, Białystok, Poland; 5 Department of Pediatrics, University Clinical Hospital in Opole, Opole, Poland; 6 Department of Pediatrics, Institute of Medical Sciences, University of Opole, Opole, Poland; 7 Department of Paediatric Diabetology and Paediatrics, Medical University of Warsaw, Warsaw, Poland; 8 Medical Research Agency, Warsaw, Poland; 9 Department of Endocrinology and Diabetology, Children’s Memorial Health Institute, Warsaw, Poland; 10 Department of Pediatrics, Endocrinology and Diabetology, Pomeranian Medical University in Szczecin, Szczecin, Poland; 11 Department of Pediatrics, Pediatric Endocrinology and Diabetes, Medical College University of Rzeszów, Rzeszów, Poland; 12 Department of Children’s Diabetology and Lifestyle Medicine, Medical University of Silesia, Katowice, Poland; 13 Department of Clinical Pediatrics, Faculty of Medical Sciences, University of Warmia and Mazury in Olsztyn, Olsztyn, Poland; 14 Department of Pediatrics, Diabetology and Endocrinology, Medical University of Gdańsk, Gdańsk, Poland; 15 Department of Pediatric Endocrinology, Jagiellonian University Medical College, Kraków, Poland; 16 Department of Pediatric Diabetes, Auxology and Obesity, Poznań University of Medical Sciences, Poznań, Poland; 17 Department of Pediatrics, Endocrinology, Diabetology and Metabolic Diseases, Wrocław Medical University, Wrocław, Poland; 18 Department of Children Endocrinology and Diabetology, Regional Polyclinical Hospital, Kielce, Poland

**Keywords:** diabetes mellitus, diabetic ketoacidosis, epidemiology, nationwide active surveillance, pandemic (COVID19)

## Abstract

**Aims:**

Global data show an increase in the rate of diabetic ketoacidosis (DKA) at type 1 diabetes (T1D) onset during the COVID-19 pandemic, but its effect on severity remain unclear. This study assessed pre-pandemic, pandemic, and post-pandemic DKA frequency in Polish children with new-onset T1D and evaluated the pandemic’s impact on DKA incidence and severity.

**Methods:**

This multicenter national retrospective study collected an anonymized list of all new-onset T1D cases in Polish children (<18y.o.) diagnosed based on clinical presentation (diabetes diagnosed based on random blood glucose >=200mg/dl and symptoms, no suspicion of other type of diabetes).

**Results:**

We analyzed 7192 children with a new-onset diabetes, and after applying the predefined inclusion and exclusion criteria, 6543 cases were ultimately included in the final analysis. Overall, 54.5% presented with DKA. Frequency increased from 47.9% in 2019 to 58.6% in 2020 (p<0.0001) and decreased to 54.0% in 2022. Observed pandemic DKA (56.8%) exceeded model predictions (47.5%; p=0.0041). The additional impact of COVID-19 was estimated at a +9.4 percentage-point increase, with a 95% CI of +9.1 to 9.6%, with DKA predicted and observed rates converging by the end of the pandemic. During the pandemic, children were also more likely to present with moderate or severe DKA compared with the rest of the study period, with a subsequent decrease in severity after the pandemic.

**Conclusions:**

DKA frequency increased across the study period, with a peak during the pandemic. This findings should be interpreted in the context of global epidemiological data, where the prevalence of DKA at diagnosis of type 1 diabetes remains high and varies widely between countries, reaching around 50% in some populations. Therefore, coordinated country-level actions aimed at improving awareness of early diabetes symptoms are needed to reduce the persistently high rate of DKA.

## 1 Introduction

Diabetic ketoacidosis (DKA) is a serious, potentially life-threatening complication of diabetes, characterized by hyperglycemia, ketosis, and metabolic acidosis. It results from insulin deficiency and elevated levels of counterregulatory hormones, such as glucagon, catecholamines, cortisol, and growth hormone (1–3). If left untreated, it can lead to altered mental status, drowsiness, focal neurological deficits, cerebral edema, and even death.

Individuals with new-onset type 1 diabetes (T1D) are among those most susceptible to developing DKA. The probability of DKA accompanying T1D diagnosis varies across countries and regions (4) and has been demonstrated to be affected by T1D screening and informational campaigns. Other biological factors, such as age, may also increase DKA risk at diagnosis, with the youngest children and adolescents especially prone to this potentially life-threatening complication (5).

During the peak pandemic period (as evidenced by COVID-19 incidence and mortality), scheduled admissions to pediatric diabetes departments declined, while emergency hospitalizations increased (6, 7). The gradual implementation of remote medical consultations posed additional challenges, particularly for children presenting with early symptoms of T1D. The nonspecific nature of these symptoms, combined with the lack of in-person medical examinations, potentially led to delayed diagnoses and an increased risk of DKA (6). To date, there is no systematic nationwide evaluation of the increase in DKA incidence and severity at the onset of T1D, particularly during and after the COVID-19 pandemic. The temporal association of healthcare overload during the pandemic and increased delays of T1D diagnosis and DKA risk is not fully known (8).

## 2 Materials and methods

### 2.1 Population

All pediatric centers for pediatric diabetes, corresponding to 16 administrative regions (voivodeships) in Poland and representing the entire Polish pediatric population, were invited to participate in a multicenter retrospective study. The study design was a retrospective chart review. As such, it was exempted from Bioethical Committee review. We followed the principles of the Declaration of Helsinki and protected patients’ confidentiality. We retrieved data on all new-onset T1D cases diagnosed between January 2019 and December 2022.

Each participating center received a uniform electronic spreadsheet to be filled with anonymized data on each new‐onset T1D case hospitalized in that unit, together with the following data: sex, age, date of diagnosis, place of living (voivodeship; urban or rural), and T1D and DKA diagnosis criteria, including: blood glucose concentration on admission, venous pH, blood gases at admission, and blood or urine ketones. C-peptide and antibodies measured as diagnostic standards (ICA, anti-GAD, ZnT8, IA2, IAA) were used to exclude other forms of diabetes, but were not systematically extracted for analysis in this manuscript.

From this population, all cases that met the following criteria were included in the study:

- age at diagnosis between 0 and 18 years of age,
- permanent residence in Poland,
- T1D diagnosed at symptomatic stage (symptoms + random blood glucose ≥200 mg/dl),
- available admission pH measurements (with or without serum bicarbonate concentration).

Exclusion criteria comprised:

- T1D diagnosed on criteria other than symptoms and random blood glucose ≥200 mg/dl,
- different type of diabetes,
- age at diagnosis >18 y.o.,
- living abroad,
- other missing data, such as date of birth, sex, or blood gases.

We used the DKA diagnostic criteria per the ISPAD 2022 guidelines: hyperglycemia [blood glucose >11 mmol/L (200mg/dL)], venous pH <7.3 or serum bicarbonate <18 mmol/L, and ketonemia (blood β-hydroxybutyrate ≥ 3 mmol/L), or moderate or large ketonuria (5). DKA severity was stratified according to the ISPAD-2022 guidelines, as:

- mild: venous pH <7.3 or serum bicarbonate <18 mmol/L,
- moderate: pH <7.2 or serum bicarbonate <10 mmol/L,
- severe: pH <7.1 or serum bicarbonate <5 mmol/L.

As part of the sensitivity analysis, we also considered DKA defined by the ISPAD 2018 guidelines, which were in effect for part of the study period. According to these guidelines, DKA was defined as serum bicarbonate < 15 mmol/L (9), with other criteria identical to those in the 2022 guidelines.

Ketone assessment, with urine assay being the most common and part of routine practice in Poland throughout the observation period, was not always recorded in the medical records, resulting in missing data that could not be retroactively traced or retrieved. Therefore, the DKA criteria were limited to blood gas results, which were sufficient for DKA classification. A summary of cases with unavailable blood gas results on admission was provided.

### 2.2 Data processing and analysis

The fraction of DKA was computed for each region (voivodship), age group (0-4, 5-9, 10-14, 15–18 y.o.), and sex (male, female) for monthly, seasonal (defined by meteorological seasons), and annual summaries. The incidence of DKA was computed for each region and year. DKA classification was based on blood gas pH. Patients with an available pH were included into the analysis of DKA rates, whereas those without pH data were reported as missing. When available, HCO_3_^-^ values were used to support severity classification.

The DKA rate changes across seasons and subgroups were analyzed using the Chi-square test with Benjamini-Hochberg correction for multiple comparisons, and with joinpoint log-Poisson regression models with a lag-1 autoregressive component (Joinpoint Trend Analysis Software, NCI, Rockville, MD, USA). Sensitivity analyses were performed to assess the impact of missing blood gas measurements by comparing DKA rates calculated among patients with available pH values with estimates obtained when cases with missing blood gas data were included in the denominator. The covariates (regions, sex, age-groups) were evaluated using simple log-Poisson regression models.

The COVID-19 pandemic period was defined following the Polish government’s statement as between March 20^th^, 2020, and May 16^th^, 2022 (10). To estimate the excess burden of DKA attributable to the pandemic, we applied synthetic control method through Bayesian time-series forecasting. Daily counts of newly diagnosed T1D cases and DKA episodes were aggregated and smoothed using rolling windows of 28 and 365 days (1-day step), and pre-pandemic period was used to train forecast under the Prophet framework, which implements a Bayesian additive regression model with linear trend and multiplicative seasonal component. This approach has been previously successfully applied for the epidemiological and healthcare time-series forecasting, including modeling of COVID-19-specific effects (11). Forecast uncertainty was estimated using Prophet’s posterior predictive simulations and expressed as 95% uncertainty intervals. The observed and estimated DKA rates (fractions occurring within defined rolling windows), were tested using Poisson two-ratio tests. Periods during which observed counts significantly exceeded model-based expectations were identified using a significance threshold of p<0.05. As the forecasting analysis was intended to estimate excess DKA burden rather than perform formal hypothesis testing across multiple independent endpoints, multiple-testing correction was not applied. We compared the DKA rates during the COVID pandemic using a synthetic control method through statistical analyses performed in Python 3.11 using the Prophet and SciPy libraries, and the code to reproduce these analyses is publicly available on Zenodo.

## 3 Results

**Figure 1:**
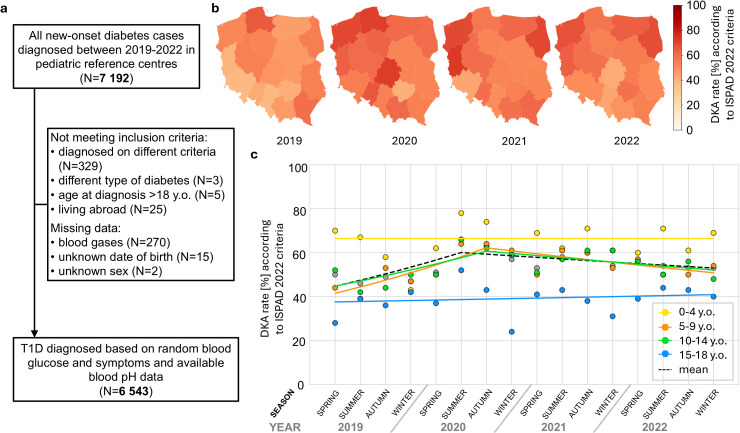
**(a)** Study flow diagram – new-onset diabetes cases reported in 2019-2022, exclusions, and final cohort. **(b)** Choropleth maps of DKA rate (%) at T1D onset by voivodeship in 2019-2022 (ISPAD-2022 criteria); darker color indicates higher DKA proportion; in łódzkie voivodships, DKA rate peaked in first year of pandemic (p=0.0063), while in lubuskie and świętokrzyskie it peaked by the end of pandemic (p=0.0301 and 0.0463, respectively). **(c)** Age-specific seasonal DKA rates from 2019 to 2022 (ISPAD-2022 criteria) from 0-4, 5-9, 10-14, and 15–18 y.o.; points show seasonal values, lines show rate trends (age-grouped and overall). y.o. – years old; T1D – type 1 diabetes; DKA – diabetic ketoacidosis.

In the analyzed period, 7192 new-onset diabetes cases were reported, with most diagnosed in 2021 (N = 1836; 28.06%). After exclusion of missing data, non-Polish citizens, other types of diabetes, and non-symptomatic T1D, the final cohort comprised 6543 cases (**Figure 1a**). Among them, 3564 (54.47%) cases were male, and the mean age at diagnosis was 8.55 ± 3.75 years. The mean pH and HCO_3_^-^ concentrations on admission were 7.28 ± 0.15 and 15.55 ± 7.14 mmol/L, respectively. 3566 (54.50%) new-onset T1D symptomatic cases presented with DKA according to the ISPAD-2022 criteria. The overall DKA rate was similar between boys and girls (ISPAD-2022, N boys=1931, N girls=1635, p=0.5693; ISPAD-2018, N boys=1644, N girls=1428, p=0.1446). DKA rate was higher in the countryside (ISPAD-2022, 56.11% vs. 53.43%, p=0.0333). Group characteristics stratified by DKA presence at onset are summarized in **Table 1** and **Supplementary Table 1**. Region-specific incidence rates were summarized in **Table 2**.

**Table 1:** Comparison of the population with DKA and without DKA diagnosed according to the ISPAD-2022 and 2018 criteria.

Variable	ISPAD 2022 criteria		ISPAD 2018 criteria	
	DKA N = 3566 (54.40)	Non-DKA N = 2977 (45.50)	DKA N = 3072 (46.95)	Non-DKA N = 3471 (53.05)
Nominal	N (%)			
Sex				
Male	1931 (54.17)	1633 (45.81)	1644 (46.12)	1920 (53.86)
Female	1635 (54.87)	1344 (45.10)	1428 (47.92)	1551 (52.05)
Habitation				
City	2141 (53.43)	1865 (46.54)	1849 (46.14)	2157 (53.83)
Countryside	1423 (56.11)^3^	1112 (43.85)	1222 (48.19)^1^	1313 (51.77)^2^
Year				
2019	692 (47.92)	751 (52.01)	583 (40.37)	860 (59.56)
2020	980 (58.61)	691 (41.33)	849 (50.78)	822 (49.16)
2021	1034 (56.29)	802 (43.66)	896 (48.78)	940 (51.17)
2022	860 (53.95)	733 (45.99)	744 (46.68)	849 (53.26)
Continuous	Mean ( ± SD)			
Age [years]	8.31 (4.27)	9.44 (4.21)	8.32 (4.30)	9.27 (4.21)
pH	7.19 (0.14)	7.39 (0.04)	7.16 (0.14)	7.39 (0.04)
HCO_3_^-^ [mmol/l]	10.08 (4.85)^6^	22.10 (2.30)	8.98 (4.37)^4^	21.27 (2.91)^5^

SD, standard deviation; ISPAD, The International Society for Pediatric and Adolescent Diabetes; DKA, diabetic ketoacidosis; non-DKA, absence of diabetic ketoacidosis.

Due to unavailable or below the detection range HCO_3_^-^ concentration data, certain cases were defined as DKA if they fulfilled only 1 criterion according to ISPAD guidelines (pH<7.3 in both 2018 and 2022).

^1^ N = 3210, as for 1 person from Śląskie voivodeship data was missing

^2^ N = 3331, as for 1 person from Świętokrzyskie voivodeship data was missing

^3^ N = 3703, as for 2 persons from Śląskie and Świętokrzyskie voivodeship data was missing

^4^ N = 2904

^5^ N = 3332

^6^ N = 3398

P-values for comparisons of DKA rates between sex and place-of-residence subgroups:

• ISPAD 2022 DKA rate in males vs. females: p=0.5693

• ISPAD 2022 DKA rate in city vs. countryside inhabitants: p=0.0333

• ISPAD 2018 DKA rate in males vs. females: p=0.1446

• ISPAD 2018 DKA rate in city vs. countryside inhabitants: p=0.1057

Overall comparisons of DKA rates across study years:

• ISPAD 2022: p<0.0001

• ISPAD 2018: p<0.0001

Pairwise comparisons of DKA rates between study years according to ISPAD 2022 guidelines:

• 2019 vs. 2020: p<0.0001

• 2019 vs. 2021: p<0.0001

• 2019 vs. 2022: p=0.0009

• 2020 vs. 2021: p=0.1636

• 2020 vs. 2022: p=0.0073

• 2021 vs. 2022: p=0.1709

Pairwise comparisons of DKA rates between study years according to ISPAD 2018 guidelines:

• 2019 vs. 2020: p<0.0001.

• 2019 vs. 2021: p<0.0001

• 2019 vs. 2022: p=0.0005

• 2020 vs. 2021: p=0.2354

• 2020 vs. 2022: p=0.0191

• 2021 vs. 2022: p=0.2202

**Table 2:** Regional characteristics of the study population by voivodeship.

Voivodeship	Newly diagnosed PwT1D	Sex	Habitation	Diagnosis per year				DKA 2018	DKA 2022	Age [years]	pH	HCO3-[mmol/l]	
	N (%)	N (%)	N (%)	N (%)				N (%)	N (%)	Mean ( ± SD)	Mean ( ± SD)	Mean ( ± SD)	N^#^
				Incidence per 100,000 children living in the respective voivodeship									
		M	City	2019	2020	2021	2022						
dolnośląskie	442 (6.76)	254 (57.47)	284 (64.25)	105 (7.28)	113 (6.76)	100 (5.45)	124 (7.78)	173 (39.14)	207 (46.83)	8.79 (4.41)	7.29 (0.14)	17.85 (6.15)	415
				22.92	24.22	24.93	33.42						
kujawsko-pomorskie	208 (3.18)	113 (54.33)	129 (62.02)	42 (2.91)	57 (3.41)	61 (3.32)	48 (3.02)	109 (52.40)	116 (55.77)	8.73 (4.15)	7.24 (0.18)	15.46 (8.64)	205
				14.29	16.78	18.83	15.01						
lubelskie	363 (5.55)	200 (55.10)	228 (62.81)	80 (5.54)	91 (5.45)	119 (6.48)	73 (4.58)	157 (43.25)	190 (52.34)	8.94 (4.36)	7.29 (0.14)	15.98 (6.31)	355
				21.97	26.19	26.19	26.19						
lubuskie	126 (1.93)	70 (55.56)	97 (76.98)	23 (1.59)	30 (1.80)	37 (2.02)	36 (2.26)	64 (50.79)	78 (61.90)	8.17 (3.78)	7.30 (0.14)	14.30 (7.12)	124
				26.42	26.42	26.42	26.42						
łódzkie	422 (6.45)	230 (54.50)	261 (61.85)	94 (6.51)	100 (5.98)	114 (6.21)	114 (7.16)	196 (46.45)	225 (53.32)	8.92 (4.18)	7.29 (0.14)	14.94 (7.17)	421
				24.29	26.27	29.54	30.37						
małopolskie	599 (9.15)	319 (53.26)	300 (50.08)	123 (8.52)	146 (8.74)	192 (10.46)	138 (8.66)	271 (45.24)	326 (54.42)	8.81 (4.24)	7.30 (0.14)	15.06 (7.21)	595
				19.5	22.49	30.88	22.76						
mazowieckie	947 (14.47)	523 (55.23)	626 (66.10)	230 (15.94)	290 (17.35)	234 (12.75)	193 (12.12)	435 (45.93)	487 (51.43)	8.74 (4.19)	7.27 (0.15)	16.00 (7.31)	849
				23.27	28.6	26.81	24						
opolskie	145 (2.22)	73 (50.34)	85 (58.62)	31 (2.15)	38 (2.27)	42 (2.29)	34 (2.13)	70 (48.28)	80 (55.17)	9.03 (4.11)	7.26 (0.13)	15.40 (7.22)	144
				19.89	25.04	27.09	23.14						
podkarpackie	365 (5.58)	207 (56.71)	152 (41.64)	89 (6.17)	83 (4.97)	102 (5.56)	91 (5.71)	172 (47.12)	210 (57.53)	9.23 (4.38)	7.28 (0.16)	14.88 (7.54)	362
				24.31	23.34	29.02	26.88						
podlaskie	248 (3.79)	131 (52.82)	136 (54.84)	53 (3.67)	45 (2.69)	82 (4.47)	68 (4.27)	139 (56.05)	159 (64.11)	9.01 (4.42)	7.28 (0.14)	13.62 (6.87)	248
				29.47	28.54	43.42	40.28						
pomorskie	456 (6.97)	246 (53.95)	231 (50.66)	57 (3.95)	119 (7.12)	147 (8.01)	133 (8.35)	244 (53.51)	281 (61.62)	8.61 (4.49)	7.26 (0.15)	14.96 (6.26)	397
				18.93	29.18	34.65	30.92						
śląskie	738 (11.28)	390 (52.85)	569* (77.10)	176 (12.20)	203 (12.15)	187 (10.19)	172 (10.80)	313 (42.41)	344 (46.61)	8.83 (4.30)	7.29 (0.15)	17.54 (6.56)	738
				24.69	26.89	27.93	27.42						
świętokrzyskie	221 (3.38)	118 (53.39)	111* (50.23)	58 (4.02)	54 (3.23)	70 (3.81)	39 (2.45)	90 (40.72)	107 (48.42)	9.03 (3.82)	7.30 (0.14)	16.45 (6.68)	207
				32.64	28.96	38.91	25.1						
warmińsko-mazurskie	213 (3.26)	116 (54.46)	131 (61.50)	55 (3.81)	46 (2.75)	65 (3.54)	47 (2.95)	107 (50.23)	123 (57.75)	8.87 (4.29)	7.26 (0.15)	15.60 (6.44)	211
				22.53	18.4	27.35	21.4						
wielkopolskie	760 (11.62)	392 (51.58)	440 (57.89)	165 (11.43)	184 (11.01)	210 (11.44)	201 (12.62)	369 (48.55)	445 (58.55)	8.74 (4.36)	7.28 (0.14)	14.37 (7.26)	760
				24.39	27.07	31	30.09						
zachodnio-pomorskie	290 (4.43)	182 (62.76)	226 (77.93)	62 (4.30)	72 (4.31)	74 (4.03)	82 (5.15)	163 (56.21)	188 (64.83)	8.86 (4.33)	7.25 (0.17)	14.07 (8.28)	290
				22.77	25.28	26.17	32.74						

PwT1D, persons with type 1 diabetes; SD, standard deviation; M, Male; DKA, Diabetic Ketoacidosis.

^*^ missing habitation data for 1 person from śląskie and 1 from świętokrzyskie voivodeship

^#^ for some patients the HCO3- concentration data was missing, in those cases DKA was diagnosed based on venous pH according to the ISPAD guidelines from 2018 and 2022 (DKA diagnosed if pH <7.3)

DKA frequency changed significantly over the years [2019: 47.9% (47.8-48.0%), 2020: 58.6% (58.6-58.6%), 2021: 56.3% (56.2-56.4%), 2022: 54.0% (53.8-54.1%), p<0.0001], with a significant increase between 2019 and 2020–2021 (p<0.0001) and decrease in DKA episodes between 2020 and 2022 (p=0.0073). The trend remained significant under the sensitivity analysis using the ISPAD-2018 definition (**Table 1**). We observed the highest rate of DKA in the Spring of 2020, which coincided with a strict nationwide lockdown in Poland (**Figure 1**). Moreover, for some voivodeships, DKA rates peaked in the first pandemic year (łódzkie voivodeship, p=0.0063, **Figure 1b**), while others at the end of the pandemic (świętokrzyskie and lubuskie, p=0.0463 and 0.0301, **Figure 1b**).

Trends in seasonal DKA rates at T1D symptomatic onset, by age group, are presented in **Figure 1c**. With the 5–9 y.o. group as the reference category, we observed significant differences in DKA rates for both the youngest (0–4 y.o.; N = 829, β=0.182, p<0.0001) and oldest (15–18 y.o.; N = 1195, β=-0.332, p<0.0001) age groups. Age groups 5–9 and 10–14 y.o. demonstrated a breakpoint in Autumn 2020 (p=0.0005 and 0.0250, respectively), changing from increasing DKA rate (sPC +6.98%, 95%CI 4.11-13.07%; and +5.20, 95%CI 1.61-20.05%, respectively) to a stable or decreasing pattern (sPC -2.22%, 95%CI -4.47 to -0.94%; and -1.68, 95%CI -11.09 to 0.01%, respectively). Detailed age-specific numbers of patients presenting with DKA are provided in the **Table 3**. Again, after these breakpoints, an upward trend in rate DKA (sPC +5.96%, 95%CI 3.21-12.48%; and +4.85, 95%CI 1.45-18.42%, respectively) stabilized or decreased (sPC -2.45%, 95%CI -4.92 to -1.07%; -0.59, 95%CI -6.63 to 0.53%, respectively).

**Table 3:** Age-specific number of patients presented with DKA according to the ISPAD-2022 guidelines.

Year	Season	Age range (N, %)			
		0–4 y.o.	5–9 y.o.	10–14 y.o.	15–18 y.o.
2019	Winter	60 (69.77)	71 (43.83)	76 (52.05)	13 (27.66)
	Spring	40 (66.67)	37 (39.36)	44 (41.90)	18 (39.13)
	Summer	38 (57.58)	60 (53.1)	49 (44.14)	13 (36.11)
	Autumn	27 (42.86)	57 (46.72)	71 (49.65)	18 (41.86)
2020	Winter	52 (61.90)	78 (50.32)	88 (49.72)	14 (36.84)
	Spring	50 (78.13)	62 (63.92)	84 (65.63)	16 (51.61)
	Summer	53 (73.61)	89 (64.49)	115 (61.83)	15 (42.86)
	Autumn	53 (58.89)	110 (61.45)	91 (58.71)	10 (23.81)
2021	Winter	73 (68.87)	85 (49.71)	95 (50.53)	15 (40.54)
	Spring	53 (62.35)	98 (61.25)	98 (56.98)	20 (42.55)
	Summer	67 (70.53)	77 (59.69)	92 (60.53)	16 (38.1)
	Autumn	59 (60.82)	79 (52.67)	89 (60.54)	18 (31.03)
2022	Winter	49 (59.76)	96 (57.49)	89 (55.97)	16 (39.02)
	Spring	60 (71.43)	73 (50.0)	70 (50.0)	22 (44.0)
	Summer	41 (61.19)	50 (50.0)	63 (55.75)	18 (42.86)
	Autumn	51 (68.92)	73 (54.48)	71 (47.65)	18 (40.0)

DKA, diabetic ketoacidosis; ISPAD, The International Society for Pediatric and Adolescent Diabetes; y.o. - years old.

**Figure 2:**
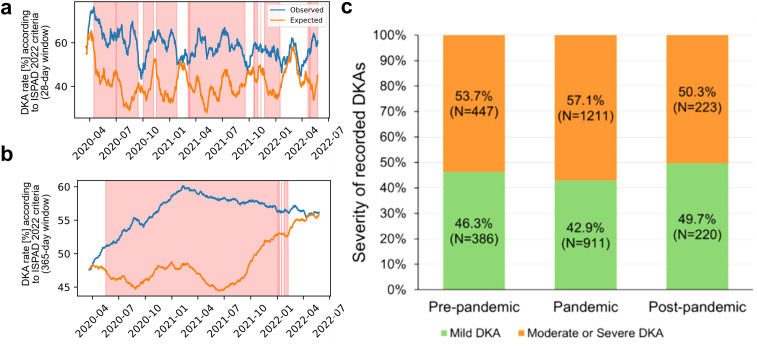
Observed (blue) and modelled (orange) DKA fraction in 28-day **(a)** and 365-day **(b)** rolling windows (using ISPAD-2022 definition), for the pandemic period (20-04–2020 to 16-05-2022); pink shading marks intervals where observed significantly exceeded modelled rate (Poisson two-ratio test p<0.05). **(c)** Severity distribution of DKA pre-pandemic, during. and post-pandemic.

The observed DKA rates in new-onset symptomatic T1D during the COVID-19 pandemic period were significantly higher than those modelled using pre-pandemic data (56.8% vs. 47.5%; p=0.0041; **Figures 2a, b**). We observed an excess of 9.4 (95%CI 9.1 to 9.6) percentage points in DKA rate between May 2020 and February 2022 (**Figures 2a,b**). Among patients presenting with DKA, the distribution of severity was 42.9% mild, 57.1% moderate or severe (**Figure 2c**). Moreover, patients diagnosed during the pandemic were more often presenting with moderate or severe DKA than during the combined pre- and post-pandemic periods (57.1% vs. 52.5%; p=0.0183). After the COVID-19 pandemic, the odds of presenting with severe or moderate DKA decreased significantly (OR = 0.76, 95%CI 0.62 to 0.93, p=0.0095).

## 4 Discussion

Our findings indicate an alarmingly high and rising frequency of DKA at T1D onset in Polish children, which mirrors and even exceeds international trends observed during the COVID-19 pandemic (12, 13). More than half of new-onset symptomatic cases of T1D in Poland during 2019–2022 presented with DKA, increasing from 47.9% in 2019 to 58.6% in 2020, and then decreasing to 54% in 2022. Notably, the surge in DKA coincided with the pandemic. The DKA rate observed during the COVID-19 pandemic was significantly higher than the available forecasts by an average of 9.4 percentage points. The post-pandemic DKA rates stabilized and even decreased significantly in some groups (e.g., children aged 5–14).

In this study, we extended the coverage to approximately 100% of the Polish pediatric population and collected pre- and post-pandemic data (14). It allows us to adopt a clinical and practical perspective and identify the most disruptive phase of the pandemic. Joinpoint analysis presented breakpoints for DKA trends in late 2020. In our forecasts, by mid-2022, COVID-19 had a limited impact on public life and healthcare services, with most restrictions already lifted (15). By the end of the official pandemic period, DKA incidence remained high at 54%, with some stabilization and a decrease, but no return to the pre-pandemic baseline​. This sustained post-pandemic plateau at an alarmingly high level underscores a serious public health concern, given the well-documented acute dangers and long-term sequelae of DKA (e.g., cerebral edema, neurocognitive impairment, and poor glycemic control) (5)​.

Between 2018 and 2022, the ISPAD criteria for diagnosing DKA were updated. In the ISPAD-2018 guidelines, DKA was defined by the presence of hyperglycemia (blood glucose >200 mg/dL), venous pH <7.3, and/or serum HCO_3_^-^ concentration <15 mmol/L. The revised ISPAD-2022 guidelines introduced a slightly higher threshold for HCO_3_^-^ concentration (<18 mmol/L) as an additional diagnostic criterion, along with a more detailed classification of DKA severity. Although the ISPAD-2022 criteria increased the number of DKA diagnoses (more sensitive criteria), the high frequency of DKA remained consistent across definitions. This suggests that changes in diagnostic thresholds alone do not explain the observed increase. A potential limitation of this study is the lack of blood gas measurements in some patients, as DKA classification required admission pH or bicarbonate values. To evaluate whether this could influence the observed trends, we performed additional analyses incorporating patients without blood gas data. Sensitivity analyses confirmed that the observed increase in DKA frequency remained unchanged.

Our data should be contextualized within global trends in DKA at T1D onset. Even before 2020, the prevalence of DKA at diagnosis was rising gradually worldwide (about 1–2% increase per year) (3, 4, 15)​. During the first two years of the COVID-19 pandemic, this rise was markedly exacerbated across many countries (15–20)​. An international study spanning 13 national registries reported that the rate of DKA at onset jumped to ~39% in 2020 and 38.9% in 2021 – significantly higher than the ~32% predicted from pre-2020 trends (p<0.0001) (15)​. This pattern was echoed in numerous regional reports: for example, in the USA, the UK, Brazil, and others, significant increases in new-onset DKA were noted during the early pandemic waves (19–26). Large European registries report that approximately 25–40% of children with newly diagnosed type 1 diabetes present with DKA. Data from the German–Austrian DPV registry indicate DKA in about one quarter of cases, with severe DKA in nearly 9%. Importantly, several studies have demonstrated an increase in DKA frequency during the COVID-19 pandemic, which has been attributed to delayed medical consultations and reduced access to healthcare services (27, 28). Italy was a notable early exception, with one report finding no immediate rise in DKA during the first wave, however, longer-term data from Italy later showed an upward trend (25). By the second pandemic year, nearly all studies reported higher DKA incidence than in the pre-pandemic period (18). Crucially, this problem has not entirely resolved with the waning of the COVID-19 pandemic. In our study and elsewhere, DKA rates remain high, even post-pandemic. For instance, a pediatric center in Turkey observed that although DKA incidence declined after the initial COVID-19 pandemic peak, it remained significantly higher than pre-2020 levels in 2022–2023 (26)​. This sustained high incidence – over 50% in Poland versus an ~27% average worldwide pre-2020 (29, 30)​ – highlights an ongoing public health issue requiring concerted action.

While increased DKA incidence during COVID-19 has been widely reported, data describing post-pandemic persistence at a nationwide level remain scarce. Several factors likely underlie both the initial spike and persistence of high DKA rates. Diagnostic delays emerged as a key contributor during the pandemic (29). Caregivers were often reluctant or unable to seek in-person medical attention for early diabetes symptoms due to lockdowns, fear of SARS-CoV-2 exposure, and overwhelmed healthcare systems​. Young children with new-onset T1D often present with non-specific symptoms that can be overlooked without a physical exam; in the context of remote consultations and reduced clinical contact, subtle signs might have gone unrecognized​. In addition, higher DKA rates in rural areas may reflect reduced healthcare accessibility. This scenario undoubtedly led to progression from mild hyperglycemia to DKA before diagnosis. Our data show that children diagnosed during the pandemic had higher odds of presenting with moderate or severe DKA compared to those diagnosed before or after the pandemic. Internationally, a correlation has been noted between the severity of the pandemic and DKA risk: regions with stricter lockdowns and higher COVID-19 mortality experienced greater increases in DKA, likely reflecting reduced healthcare access and heightened public fear. COVID-19 infection itself might have contributed in part, e.g., via increased metabolic stress or islet autoimmunity. However, direct biological links remain inconclusive (31).

The breakpoint observed in Spring 2020 coincided with the first nationwide lockdown in Poland, characterized by school closures and restricted access to outpatient care.

Our findings may suggest that system-level factors, particularly healthcare accessibility and diagnostic timing, play a critical role in reducing the DKA rate. The pandemic essentially stress-tested the system and exposed existing vulnerabilities in the timely diagnosis of pediatric diabetes. Unfortunately, the system-wide issues were not resolved with the end of the COVID-19 pandemic, which might result in persistently high DKA rates. For example, many families and primary care providers have become accustomed to telehealth or are still catching up on delayed care (24). Likewise, any backlog or strain in primary care and pediatric referrals can prolong the window in which pre-clinical diabetes silently progresses to DKA. Addressing these factors is critical to resolve the post-pandemic high rate of DKA at T1D diagnosis.

Historically, Poland’s DKA frequency has been on the higher side (about one-third of cases) even in the late 2010s (32)​. This contrasts with some countries that have maintained much lower DKA proportions through strong awareness and early-diagnosis practices. For instance, Sweden, despite a much higher reported incidence of T1D, reports DKA in only ~20% of new cases. Other high-income countries average around 20–30% DKA at diagnosis (33). The need for healthcare policy changes is critical. A systematic review of diabetes awareness campaigns worldwide concluded that such interventions can effectively reduce DKA incidence (34, 35). An example from Italy, which historically had DKA rates of 35–40%, provides compelling evidence that targeted awareness campaigns may significantly reduce DKA incidence. One large-scale campaign in Parma, Italy, reportedly reduced the incidence of pediatric DKA from 78% to 12.5% within the campaign area. Likewise, in the UK, the “4T’s” diabetes awareness campaign (Toilet, Thirsty, Tired, Thinner) and similar initiatives have been associated with measurable drops in DKA rate through caregiver symptom recognition (34).

Beyond education, proactive screening for presymptomatic T1D is emerging as a powerful strategy to prevent DKA at onset. The pioneering initiatives have demonstrated that it is possible to identify children at high risk of T1D before they develop symptoms, and thereby ensure close monitoring and timely treatment initiation. The Fr1da study is a landmark example, demonstrating that children diagnosed at a presymptomatic stage almost never present with DKA at T1D onset (36). In fact, DKA occurred in <5–10% of children who were identified in stage 1 or 2 T1D, compared to ~20–50% among children diagnosed in the usual symptomatic way​. Building on this success, several multicenter screening initiatives have been launched. The Global Platform for the Prevention of Autoimmune Diabetes (GPPAD) has been screening infants for high genetic risk of T1D in multiple European countries, aiming to both study prevention and ensure early detection (37). Programs such as INNODIA and TrialNet are enrolling relatives of individuals with T1D to monitor for early-stage disease (38, 39). Additionally, new population-based screening studies, such as the ongoing Edmonton and Fr1da extensions (40), and the EU-funded EDENT1FI project (41), are evaluating the feasibility of broader childhood screening using genetic and autoantibody tests. Moreover, as new treatment opportunities emerge to delay T1D onset, the public health interest in T1D screening programs increases (40). It is noteworthy that Italy has become the first country to move toward nationwide T1D screening. In late 2023, the Italian Parliament enacted a law to implement general population screening for T1D (and celiac disease) in children 1–17 years old ​ (37). This unprecedented legislative step reflects the importance of reducing DKA at T1D onset as a public health priority.

Considering our findings and the global evidence, several interventions are advisable for Poland. First, a renewed public awareness campaign is imperative. Past experience from other countries suggests that a well-designed campaign (leveraging social media, schools, and pediatric clinics) about the early symptoms of diabetes could substantially reduce diagnostic delays. Messages should target both parents and healthcare providers, particularly general practitioners and emergency department staff, emphasizing classic signs (polyuria, polydipsia, weight loss, enuresis) and urging immediate fingerstick glucose testing when T1D is suspected (35). Engaging national media and organizations (e.g., professional medical or patient advocacy groups) in a coordinated “know the signs” campaign could replicate the successes seen in the UK and Italy. Such efforts have been shown to be cost-effective, given that preventing even a single DKA hospitalization can offset the campaign expenses (42)​. Importantly, in 2025, the Polish Society of Pediatric Endocrinology and Diabetology launched a national campaign (43), representing a crucial step toward improving early recognition and reducing the burden of DKA.

Second, primary care training and protocols should be strengthened. DKA prevention hinges on family clinicians and pediatricians promptly recognizing T1D. Healthcare systems can implement decision support or triage prompts to “think of diabetes” for relevant presentations (44, 45). Continuing medical education programs in Poland may focus on pediatric diabetes warning signs and highlight recent data indicating that more than half of new symptomatic T1D cases present with DKA. Strengthening undergraduate education in pediatric diabetes is also essential, as medical students’ knowledge of diabetes is often incomplete (46).

Third, healthcare accessibility must be improved, particularly in the post-pandemic landscape. This may involve ensuring that in-person pediatric appointments are readily available and that telemedicine, while convenient, is supplemented by timely physical evaluations when diabetes is a possibility. Streamlining referral pathways from primary care to specialized centers, e.g., through rapid-access to referral diabetes centers for children presenting with new-onset diabetes symptoms, could reduce delays. Our study covered all 16 voivodeships, revealing some regional and city/rural disparities. Targeted interventions in areas with the highest DKA rates are warranted (14).

This study has several important strengths. This is the first nationwide, multicenter study covering nearly 100% pediatric population with new-onset T1D in Poland and systematically evaluating temporal, seasonal, regional, and demographic patterns across the pre-pandemic, pandemic, and post-pandemic periods. In the context of recent European and global reports, it constitutes one of the largest nationwide analyses of DKA at T1D diagnosis in children conducted in the past decade. This approach reduces selection bias and enhances the generalizability of our findings at the national level. The use of a standardized DKA classification enhances the robustness and comparability of results. The nuanced statistical methods allowed us to move beyond simple year-to-year comparisons and to quantify excess DKA rates during periods of healthcare disruption. Several limitations should be acknowledged. The retrospective design relies on routinely collected clinical data, which limits control over data completeness and granularity, especially for ketone measurements. Although DKA diagnosis was based on standard blood gas criteria used in routine clinical practice, the lack of data on ketonemia/ketonuria limited the ability to verify ketosis in all cases. Consequently, reliance primarily on blood gas criteria may have affected the diagnostic specificity of DKA classification. We were unable to directly assess diagnostic delays, healthcare-seeking behavior, telemedicine use, socioeconomic status, parental education, or prior healthcare contacts. Changes in DKA diagnostic criteria during the study period could have modestly increased diagnostic sensitivity, thereby affecting data completeness in the not-missing-at-random scenario. Additionally, exclusion of patients due to incomplete blood gas data may have influenced the estimated DKA rates, as some excluded patients could potentially have presented without DKA, thereby reducing the denominator of the analyzed cohort. Moreover, we were unable to assess data on prior or concomitant COVID-19 infection and future studies incorporating individual infection data may provide further insight into this association. The analysis of DKA severity did not account for potential confounders such as age, sex, urban versus rural residence or region. Despite these limitations however, the data shows a steady and alarming pattern of increasing DKA necessitating continued efforts to mitigate this trend.

## 5 Conclusions

Given the life-threatening nature of DKA and its long-term impacts on children’s health, an observed high rate of DKA at new onset symptomatic T1D across all voivodeships in Poland is an important public health issue. Although the post-pandemic peak in the DKA rate has stabilized and even declined in some patient groups, it has highlighted critical gaps in the Polish healthcare system. Immediate action is needed, and other countries’ experiences, from awareness campaigns to screening trials, provide a roadmap for success.

## Data Availability

The raw data supporting the conclusions of this article will be made available by the authors, upon reasonable request, without undue reservation. The code to reproduce the analysis is available at https://doi.org/10.5281/zenodo.17985488.
