# Supplementary material for: Persistently increased frequency of diabetic ketoacidosis in new-onset type 1 diabetes in Polish children: nationwide analysis 2019–2022
Source: Front Endocrinol (Lausanne). 2026 Jun 29;17:1861694. doi: 10.3389/fendo.2026.1861694 (PMC13357116; doi:10.3389/fendo.2026.1861694)
Supplement: Supplementary file 1 [file Table1.docx]

| **Nominal** | | |
| --- | --- | --- |
| **Variable (N = 6543)** | **N (%)** | |
| **Sex** | **M** | **F** |
|  | 3564 (54.47) | 2979 (45.53) |
| **Habitation^1^** | **City** | **Country** |
|  | 4006 (61.23) | 2535 (38.74) |
| **Voivodeship**  dolnośląskie  kujawsko-pomorskie  lubelskie  lubuskie  łódzkie  małopolskie  mazowieckie  opolskie  podkarpackie  podlaskie  pomorskie  śląskie  świętokrzyskie  warmińsko-mazurskie  wielkopolskie  zachodnio-pomorskie | 442 (6.76)  208 (3.18)  363 (5.55)  126 (1.93)  422 (6.45)  599 (9.15)  947 (14.47)  145 (2.22)  365 (5.58)  248 (3.79)  456 (6.97)  738 (11.28)  221 (3.38)  213 (3.26)  760 (11.62)  290 (4.43) | |
| **Year of diagnosis**  2019  2020  2021  2022 | 1443 (22.05)  1671 (25.54)  1836 (28.06)  1593 (24.35) | |
| **Continuous** | | |
| **Variable** | **Mean (±SD)** | |
| **Age [years]** | 8.83 (4.28) | |
| **pH** | 7.28 (0.15) | |
| **HCO_3_^-^ concentration^2^ [mmol/l]** | 15.55 (7.14) | |

SD – standard deviation; M – Male; F – female

1 – N=6541 as for 2 persons from świętokrzyskie and śląskie voivodeship data was missing

2 – N=6236 as for some persons HCO_3_^-^ concentration was unavailable or was below the detection range
